# NF-κB-mediated regulation of rat CYP2E1 by two independent signaling pathways

**DOI:** 10.1371/journal.pone.0225531

**Published:** 2019-12-27

**Authors:** Qin Lin, Xiaolin Kang, Xuefeng Li, Tao Wang, Fengting Liu, Jinxue Jia, Ziqi Jin, Yongzhi Xue

**Affiliations:** Institute of Pharmacokinetics and Liver Molecular Pharmacology, Department of Pharmacology, Baotou Medical College, Baotou, Inner Mongolia, China; National Institutes of Health, UNITED STATES

## Abstract

Cytochrome P450 2E1 (CYP2E1) plays an important role in both alcohol-induced and immune-mediated liver injury. However, the mechanism underlying CYP2E1 transcriptional regulation has not been clarified. This study focused on the NF-κB-mediated transcriptional regulation of rat CYP2E1 by two independent signaling pathways in alcohol-induced and immune-mediated liver injury rat models. Male Sprague-Dawley rats were used in pharmacokinetic, molecular pharmacology, and morphology experiments. A rat model of alcohol-induced liver injury (AL) was established by feeding an ethanol-containing diet (42 g/kg/day) for 5 weeks as indicated. A rat immune-mediated liver injury (IM) model was established by the sequential injection of *bacillus Calmette-Guérin* (BCG, 125 mg/kg, once) via the tail vein after test day 21 and 10 μg/kg LPS 13 days later. HPLC, real-time PCR, western blot and ELISA analyses were performed. CYP2E1 expression was enhanced during the process of alcohol-induced liver injury (increased by 56%, *P* < 0.05) and significantly reduced during that of immune-mediated liver injury (reducedby52%, *P* < 0.05). NF-κB was activated in both the AL and IM groups (increased by 56% and76%, respectively, *P* < 0.05). Compared to those in the livers of AL model rats, the interleukin (IL)-1β, tumor necrosis factor (TNF)-α, and iNOS levels in IM model rat livers were increased (increased by 26%, 21% and 101%, respectively, *P* < 0.05). The differential changes in CYP2E1 in the processes of alcohol-induced and immune-mediated liver injury may result from the differential expression of inflammatory cytokines and iNOS after NF-κB activation, leading to the NF-κB-mediated transcriptional regulation of rat CYP2E1 by two independent signaling pathways.

## Introduction

Cytochrome P450 2E1 (CYP2E1) is a member of the CYP superfamily in the mammalian liver that undergoes dramatic changes during both alcohol-induced and immune-mediated liver injury and is involved in injury mechanisms [1–2]. Insights into the complex mechanisms of immune-mediated CYP suppression have been obtained using bacterial sepsis as an acute model of inflammation [2]. Reduced hepatic CYP expression and activity were shown to be primarily due to transcriptional suppression but might also involve posttranslational protein modifications induced by mediators produced as a consequence of inflammation and infection [3]. The proinflammatory cytokines TNF-α and IL-1β are recognized as the most potent mediators for reducing CYP activity and expression [4,5]. NF-κB may interfere with the injury process in the liver by regulating the inflammatory cytokines IL-1β and TNF-α and the nitrosative stress factor iNOS, thereby affecting the expression and metabolic activity of CYP2E1 [6,7]. The liver is also the primary site of alcohol metabolism and, as such, is the primary target of alcohol toxicity. In addition to contributing to ethanol-induced oxidative stress and liver injury [6], CYP2E1 is primarily involved in the oxidative metabolism of ethanol [5]. Many laboratories, including those of Correaet al. [8] and Buturaetal. [9], have researched the role of CYP2E1 and its upregulation by alcohol. Many previous studies utilized CYP2E1 inhibitors, CYP2E1 knockout mice, and transgenic CYP2E1 overexpression. Using a similar approach, McClain et al. [10] evaluated the role of cytokines in alcohol-induced liver injury, especially focusing on TNF-α, NF-κB, endotoxin (lipopolysaccharide, LPS), nitric oxide, and iNOS. Moreover, Morgan et al.[11] reported that the levels of cytochrome P450 enzymes were decreased by inflammation, infection, cytokines, and nitric oxide. However, the specific changes to CYP2E1 following immune-mediated and alcohol-induced liver injuries, the role and underlying mechanism of CYP2E1, and whether the injury process can be influenced by the selective regulation of CYP2E1 or CYP2E1-dependent oxidative stress have not yet been determined.

In summary, animal models of immune or alcoholic liver injury were previously applied, and different laboratories drew similar conclusions, including that inflammatory cytokines such as IL-1β and TNF-α as well as iNOS are involved in the regulation of CYP2E1; however, inter model differences regarding the contribution of CYP2E1 to metabolism and toxicity in the last decade are not well understood [12]. Therefore, two different models of *in vivo* liver injury were utilized in the same study, and pharmacokinetic and immunopharmaceutical methods were used to further explore the interactions regulated by oxidative/nitrative stress, immune responses and metabolic enzymes to reveal the transcriptional regulatory mechanisms associated with differential changes in metabolic enzymes.

## Materials and methods

### Experimental animals and reagents

Eight- to nine-week-old male Sprague-Dawley rats (body weight 210 ± 20 g, licensure: SCXK 2005–2001) were supplied by the Department of Laboratory Animal Science of Inner Mongolia University and maintained under a 12 h light/12 h dark cycle (light cycle from 06:00 to 18:00) and a regulated environment (20±1°C). The animals were housed in plastic cages and had access to food and water *ad libitum*. The animal experiments were approved by the Committee of Animal Ethics in Baotou Medical College and carried out in accordance with the requirements of Chinese National Legislation. The *Mycobacteriumbovis* BCG vaccine (60 mg, batch number: 2015–1) and chlorzoxazone (CHZ) reference substance (purity: 99.5%) were purchased from the National Institute for the Control of Pharmaceutical and Biological Products (Beijing, China). Pyrrolidine dithiocarbamate (PDTC; batch number: 851002), aminoguanidine (AG) and LPS (*Escherichia coli* LPS; serotype 055:B5) were purchased from Sigma (St. Louis, MO, USA). The cytoplasmic and nuclear protein extraction kit; bicinchoninic acid (BCA) assay kit; sodium dodecyl sulfate polyacrylamide gel electrophoresis (SDS-PAGE) preparation kit; rabbit polyclonal antibodies for CYP2E1 (catalog number: PB0186), iNOS (catalog number: EK0394), NF-κB (catalog number: BA0610), Lamin A/C (LMNA) antibody (catalog number: BA1227), and GAPDH (catalog number: BA2913); BCA protein assay kit (catalog number: AR0146), rat IL-1β enzyme-linked immunosorbent assay (ELISA) kit (catalog number: BA2913), and rat TNF-α ELISA kit (catalog number: BA0527) were obtained from Wuhan Boster Biological Engineering Limited Company. The RT-PCR Kit (catalog number: BMB05M1) was purchased from Biosystems Research Corp. (Nanjing, China). The RT-PCR primers were synthesized by Sangon Biotech Company (Shanghai, China).

### Preparation of immune-mediated and alcohol-induced liver injury animal models and animal groupings

**Fig 1:**
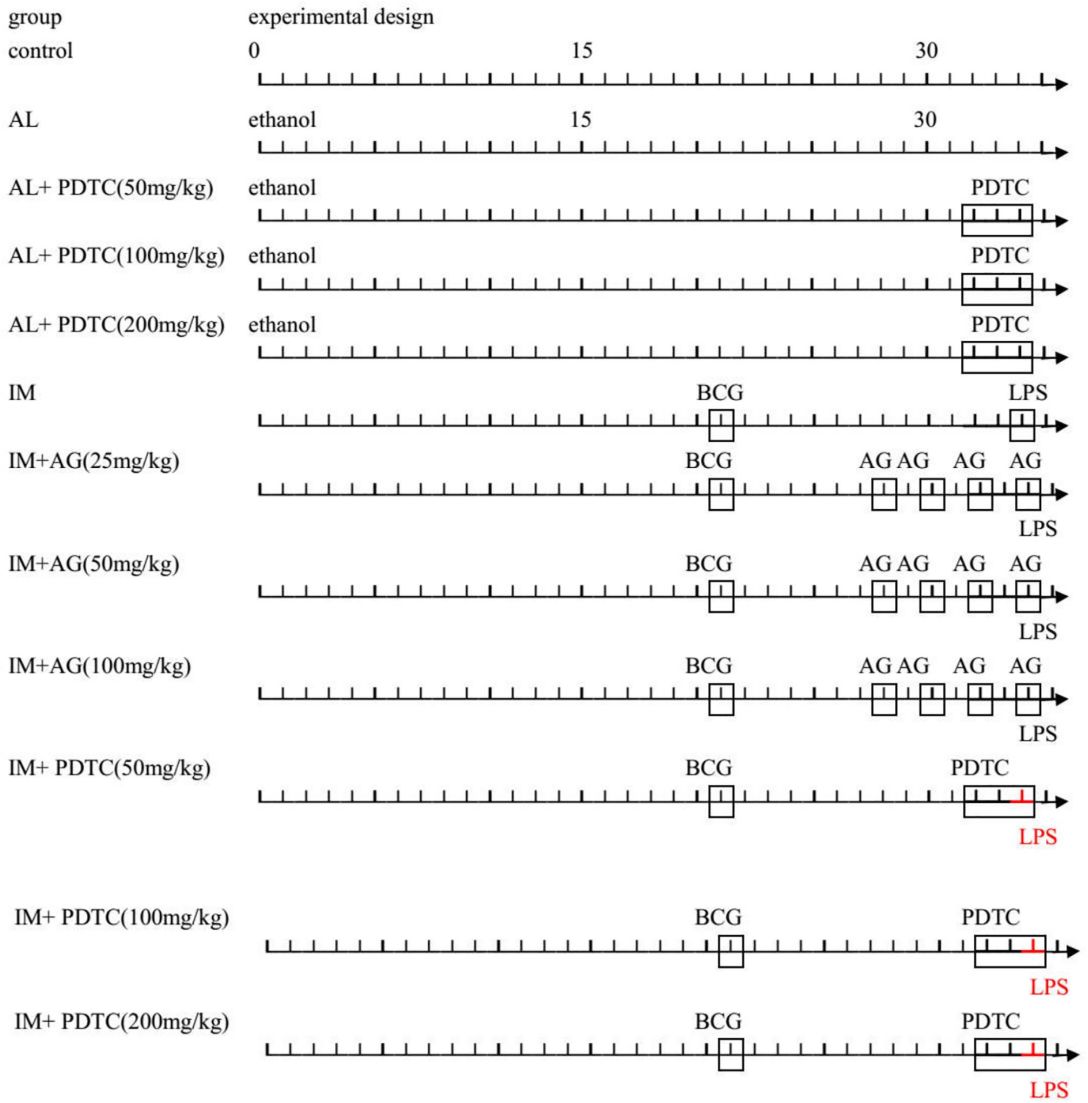
Animal groupings and experimental design.

In total, 108 male Sprague-Dawley rats were adaptively fed for two weeks and randomly divided into twelve groups of 9 rats each after weighing. Animals in the first group, the control group, were pair-fed a liquid diet in which maltose dextrin was isocalorically substituted for ethanol throughout the entire feeding period [13]. Animals in the second group, the alcohol-induced liver injury group (AL), had free access to a liquid diet in which 17% of the calories were from ethanol (3.3% v/v) for 2 days and then to a liquid diet in which 35% of the calories were from ethanol (6.7% v/v) for 5 weeks. Rats in the PDTC intervention group were intraperitoneally injected with PDTC on the 32nd, 33rd, and 34th days after ethanol feeding (50, 100, and 200 mg/kg, respectively). Animals in the sixth group, the immune-mediated liver injury group (IM), were intravenously injected via the tail vein with BCG (125 mg/kg, once) after test day 21 and with 10 μg/kg LPS13 days later. Animals in the AG intervention group were intraperitoneally injected with AG on the 7th, 9th, 11th, and 13th days after the BCG injection (25, 50, 100 mg/kg, respectively). Animals in the PDTC intervention group were intraperitoneally injected with PDTC on the 11th, 12th, and 13th after the BCG injection (50, 100, and 200 mg/kg, respectively). At the end of the experiment, three rats from each of the twelve groups were decapitated (12 h post-LPS injection). The livers were harvested and weighed, and the liver tissues were fixed in a formaldehyde-alcohol stationary liquid (formaldehyde:alcohol = 1:9) that was prepared in advance. Some tissue sections were embedded in paraffin for histopathological analysis using immunochemistry staining. Other liver tissues were snap-frozen in liquid nitrogen for western blot, real-time PCR (RT-PCR), and ELISA analyses. The remaining six rats in each of the twelve groups were tested for CYP2E1 metabolic activity by high-performance liquid chromatography (HPLC) at the end of the experiment. See Fig 1 for groupings and experimental design.

### Immunohistochemistry (IHC)

Paraffin-embedded specimens were sectioned at a 4 μm thickness according to standard histopathological techniques [14]. After deparaffinization via serial xylene baths, the samples were rehydrated via a graded ethanol series. The endogenous peroxidase activity was blocked with 3% H_2_O_2_ for 10 min. The sections were stained with a primary anti-iNOS antibody (1:200) and incubated overnight at 4 °C. Thereafter, the secondary IgG antibody (1:500) was added, and the sections were incubated for another 30 min at 37 °C. Subsequently, the sections were stained with diaminobenzidine (DAB) after incubation with a secondary antibody, followed by counterstaining with hematoxylin. Nonspecific binding was blocked by 5% normal goat serum for 10 min. Visual analysis was performed using ImageScope software (Aperio Technologies, San Diego, California, USA).

### HPLC measurement of CYP2E1 metabolic activity in rats

At the end of the experiment, rats in the control, AL, and IM groups were intragastrically administered CHZ (50 mg/kg). Blood from the angular vein (0.2 mL) was collected from the eye at 0.5, 1, 2, 3, 4, and 6 h after CHZ administration. The blood samples were placed in heparinized tubes and centrifuged at 3500 g for 5 min. The plasma was collected and stored in a refrigerator at -20°C until further analysis. According to a previously described method [14], the plasma concentration–time courses and pharmacokinetic parameters (maximum concentration, *C*_*max*_; terminal half-life, *T*_*1/2*_; peak time, *T*_*max*_; area under the plasma concentration–time profile, AUC; and elimination rate constant, *Ke*) of CHZ were measured and fitted with DAS 3.0 software (Shanghai Bojia Pharmaceutical Technology Co., Ltd.). CYP2E1 metabolic activity was assessed by determining the concentration–time curve and pharmacokinetic parameters of the probe drug CHZ [7]. The levels of CHZ metabolized to 6-hydroxychlorzoxazone (6-OHCHZ) in rat plasma samples were estimated by HPLC [15]. The CYP2E1 phenotypic index was assessed by comparing the metabolic ratios (6-OHCHZ/CHZ) of *C*_*max*_, AUC, *T*_*1/2*_, and *Ke* obtained after CHZ administration during the control and treatment phases.

### Western blot analysis of CYP2E1, iNOS, and NF-κB

The liver tissues were weighed, and liver tissue proteins were prepared according to the instructions provided in the protein extraction kit. Among them, nuclear protein was used for NF-κB detection, and liver tissue protein was used for the detection of iNOS and CYP2E1. The proteins were quantitated by the BCA assay, and equal amounts of protein (30 μg) were separated on 5–10% polyacrylamide gels and electrophoretically transferred onto polyvinylidene difluoride membranes. Nonspecific binding was blocked with tris-buffered saline containing 5% skim milk for 1 h at 25°C. The membranes were sequentially incubated with CYP2E1, iNOS, LMNA, GAPDH, or NF-κB rabbit polyclonal antibodies diluted 1:200 in blocking solution for 2 h at room temperature and then with an anti-rat HRP antibody at a 1:2000 dilution in blocking solution for 30 min at 25 °C. The blots were washed, and the proteins were visualized using an enhanced chemiluminescence method according to the manufacturer’s instructions and photographed using a gel imaging system. A GAPDH rabbit polyclonal antibody was used as the internal standard for CYP2E1 and iNOS, while an LMNA rabbit polyclonal antibody was used as the internal standard for NF-κB.

### Measurement of TNF-α and IL-1β levels

The liver tissues (0.2 g) were sliced accurately, and PBS was added (1 mL, pH = 7.4). The tissue slices were homogenized and centrifuged for 20s (3500*g*), and the supernatant was collected. According to the instructions provided in the kits, the absorbance of the sample in each well was measured by a microplate reader at 450 nm within 30 min. Wells containing only the chromogenic substrate 3,3′,5,5′-tetramethyl benzidine (TMB) were considered blank controls. The optical densities (ODs) of the standards and samples were subtracted from the ODs of the TMB control wells. The results were plotted against a standard curve, and the linear regression equation was determined. The IL-1β and TNF-α concentrations in the liver homogenates were calculated using the standard curve equation.

### Quantitative real-time RT-PCR analysis

Experiments to determine RNA concentrations were performed in 96-well plates. Total RNA was extracted using a Total RNA FAST2000 Kit. One microgram of total RNA was used for cDNA synthesis using the BioRT Two Step RT-PCR Kit (Thermo Fisher Scientific, Applied Biosystems, Grand Island, NY), and real-time PCR was carried out with 2× real-time PCR Master Mix (Thermo Fisher Scientific, Applied Biosystems, Grand Island, NY) on a 7900HT Fast Real-Time PCR system. GAPDH mRNA was used as the normalization control. The primers described by Sangon Biotech were used for CYP2E1 isoform measurements by real-time PCR. The forward primer for the amplification of CYP2E1was5′-TGGACGCTGTAGTGCATGAGATTC-3′, and the reverse primer was 5′-TCCTCGGAACACGGTGTCTCG-3′. The GAPDH primers used were 5′-AAGAAGGTGGTGAAGCAGGCATC-3′ (forward primer) and 5′-CGGCATCGAAGGTGGAAGAGTG-3′ (reverse primer). Real-time PCR analyses were carried out using the ΔΔCt method. The experiment was repeated in triplicate for each of the samples to yield a single mean sample value. The total number per group used to obtain the mean consisted of three different experiments.

### Statistical analysis

All data are presented as the mean ± standard deviation (SD) from ten independent experiments. Statistical analysis was performed using one-way analysis of variance (ANOVA) followed by Tukey’s test. *P*< 0.05 was considered statistically significant.

## Results

### Pathological manifestations in the rat livers

**Fig 2:**
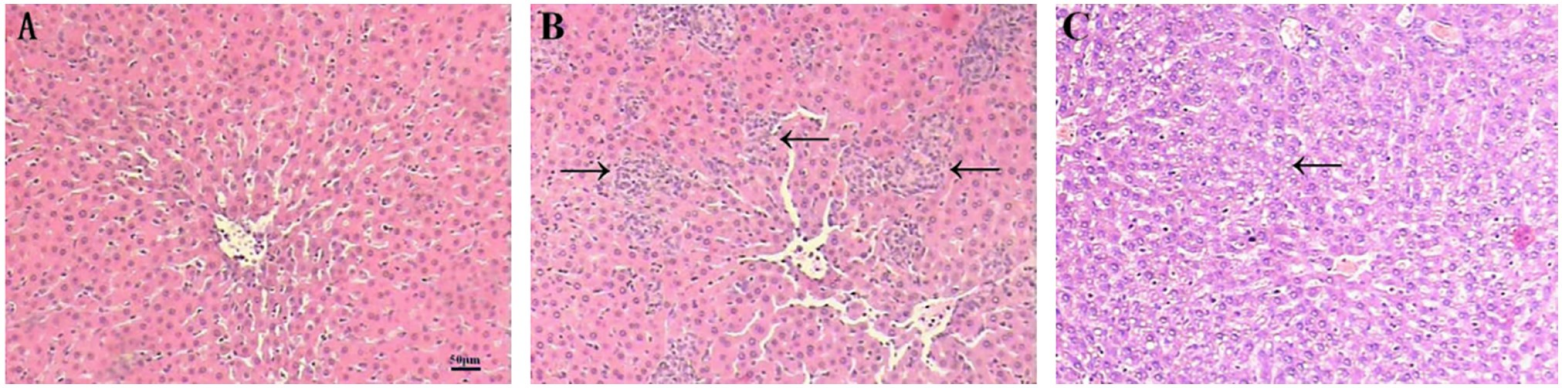
Effect of immune-mediated and alcohol-induced liver injury on histopathological changes in the rat liver. (A–C): Representative light microscopic photos showing the appearance of rat liver tissue (HE staining; original magnification ×200) for the control group (A); immune-mediated (IM) liver injury group (B, the arrows in the figure point to a granuloma mass consisting of inflammatory cells); and alcohol-induced (AL) liver injury group (C, the arrow points to hepatocyte steatosis).

After two weeks, normal rats showed no histological abnormalities, whereas rats in the IM group showed various histological changes in the liver. The liver sections from each group stained with hematoxylin and eosin (HE) were evaluated under a light microscope (Fig 2A–2C). In the normal saline (NS) control group, the hepatic lobules were structured normally and had regular and radial hepatic cords, the liver cells were uniform in size with a round nucleus and a clear cytoplasmic nucleolus, and little inflammatory cell infiltration was observed in the portal area (Fig 2A). In the IM group, the liver parenchyma and portal area were surrounded by large numbers of monocytes and lymphocytes constituting granuloma clumps of varying sizes and diffuse distribution, which resulted in an unclear hepatic cord structure. The liver cells demonstrated vacuolar degeneration (Fig 2B). Rats in the AL group exhibited obvious liver damage, unclear hepatic lobule structures, and disordered hepatic cords. The liver cell volume increased with moderate water diffuse degeneration and compressed the liver sinus. Most of the hepatic cells showed fat vacuoles of varying sizes in the cytoplasm that fused into large lipid droplets and pushed the nuclei to one side. This effect was obvious at the periphery of the hepatic lobules (Fig 2C).

### Manifestation of immunohistochemical changes in the rat livers

**Fig 3:**
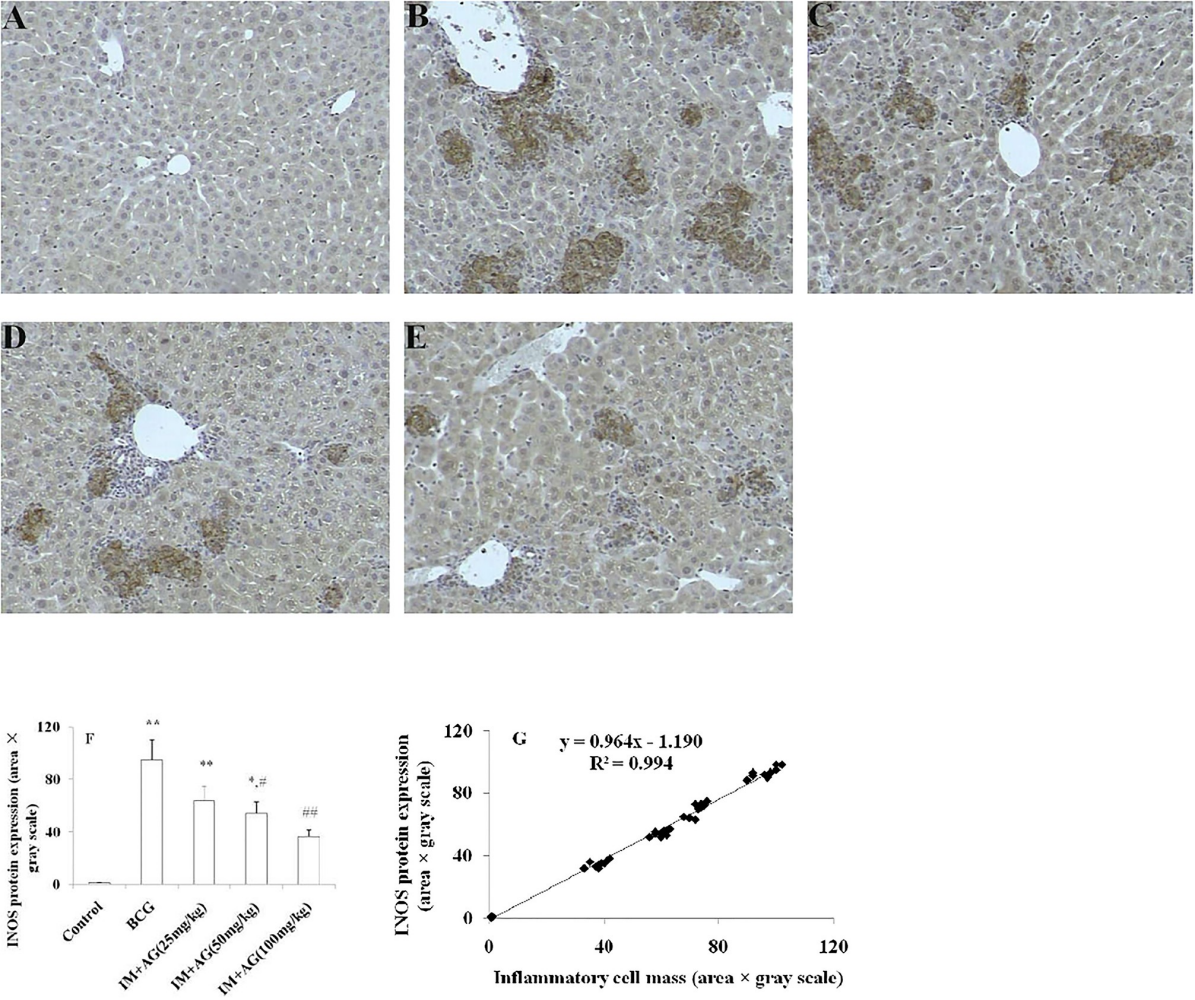
Histopathological effects of inhibiting iNOS activation in rats with immune liver injury. Immunohistochemistry demonstrated fewer iNOS cells (brown) in the AG treatment group than in the BCG group (BCG, *bacillus Calmette-Guérin*; iNOS, inducible nitric oxide synthase; and AG, aminoguanidine), as shown in Fig 3 (A-F). A shows the control group, B shows the IM group, and C-E shows BCG stimulation together with small (25 mg/kg), medium (50 mg/kg) and large doses (100 mg/kg) of aminoguanidine for intervention. The brown mass was positive for iNOS. Three low-power field of views were randomly selected for each slice, and the grayscale and area scans were performed by image analysis software (Image Pro Plus 6.0). The results are shown in Fig 3F. *P<0.05 and **P<0.01 vs the control group. ^#^*P*<0.05 and ^##^*P*<0.01 vs the IM group. Significance was determined by one-way analysis of variance (ANOVA) followed by Tukey’s test. Image grayscale gradient scanning was performed based on the intensity of the inflammatory cell mass and iNOS expression in liver tissue caused by BCG stimulation. After correlation analysis using binary variables, as shown in Fig 3G, the two were obviously positively correlated (*r* = 0.99, n = 9, *P*< 0.01).

Accompanied by inflammatory reactions, substantial iNOS induction was observed in the liver tissues of rats with immune liver injury, although no such changes were observed in rats with alcoholic liver injury. To identify the relationship between liver pathology and iNOS expression, we performed immunohistochemical staining followed by counterstaining with hematoxylin; representative immunohistochemistry results are shown. The iNOS protein expression was obviously increased in the IM group compared with that in the control group (Fig 3A and 3B); in contrast, AG treatment suppressed the expression of iNOS in a dose-dependent manner (*P*< 0.05, Fig 3C–3F). Interestingly, the localization of iNOS protein expression was consistent with that of inflammatory cell masses, and AG administration alleviated the histopathological changes in a dose-dependent manner. Image grayscale gradient scanning was performed to assess the inflammatory cell mass intensity and iNOS expression in liver tissues caused by BCG stimulation; three fields of view for each slice were selected for scanning. After correlation analysis by binary variables, as shown in Fig 3F and 3G, the inflammatory cell mass intensity and iNOS expression were obviously positively correlated (*r* = 0.99, n = 9, *P*< 0.01).

### Changes in the CYP2E1 metabolic activity in each group

**Fig 4:**
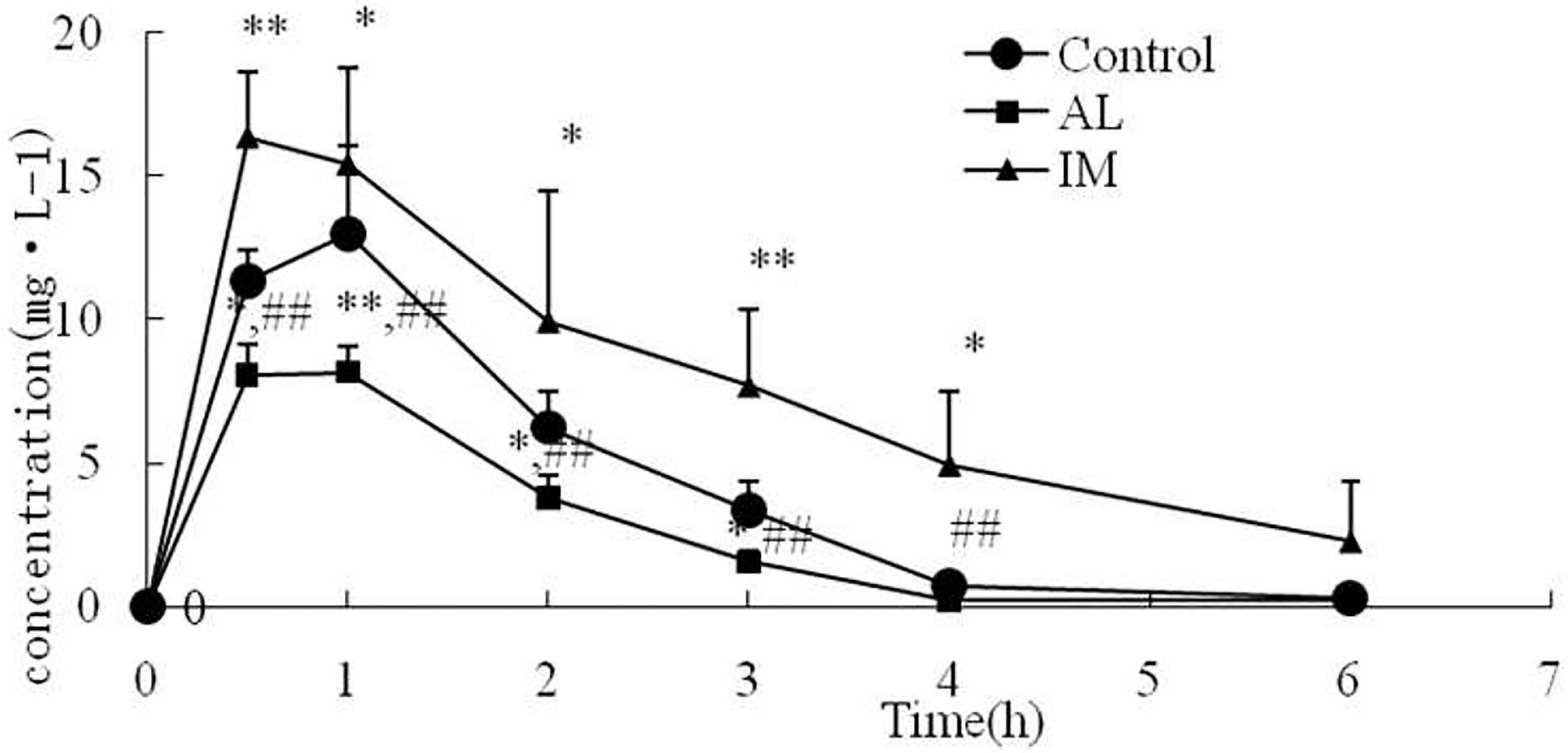
Plasma concentration–time profiles of chlorzoxazone (50 mg/kg) after oral administration to normal saline control rats, immune-mediated liver injury rats, and alcohol-induced liver injury rats. Blood samples were collected at 0.5, 1, 2, 3, 4, and 6 h post chlorzoxazone administration. Plasma chlorzoxazone levels were measured as described in the Materials and methods. Data represent the mean ± SD (n = 6).*P<0.05 and **P<0.01 vs the control group. #P<0.05 and ##P<0.01 vs the IM group. The significance was determined by one-way analysis of variance (ANOVA) followed by Tukey’s test.

Rats in the control, AL, and IM groups received a single oral dose of CHZ (50 mg/kg), a probe drug metabolized by CYP2E1. The changes in the CHZ and 6-OHCHZ plasma concentration-time curves were measured by HPLC. The plasma CHZ concentration in the IM group was higher than that in the control group at each time point. However, the plasma CHZ concentration in the AL group was lower than that in the control group at 0.5, 1, 2, and 3 h post-CHZ administration (*P*< 0.05; Fig 4). The IM group showed higher AUC, *T*_*1/2*_, and *C*_*max*_ values than the control group (*P*< 0.05). The AUC, *T*_*1/2*_, and *C*_*max*_ values in the AL group were lower than those in the IM and control groups (*P*< 0.05). *T*_*max*_ and *Ke* did not differ among the groups (Table 1).

**Table 1:** The pharmacokinetic parameters of chlorzoxazone after a single oral dose (50 mg/kg) in control, IM, and AL rats (50 mg*·*L^-1^, mean ± SD, *n* = 6).

group	n	control	AL	IM
AUC/ h·mg·L^-1^	8	26.64±4.44	16.53±0.82*^,^^##^	57.49±18.79**
*T*_*1/2*_/h	8	0.82±0.16	0.57±0.07*^,^^##^	1.64±0.32*
*C*_*max*_*/*mg·L^-1^	8	13.35±1.52	8.69±0.93*^,^^##^	18.58±1.30*
*T*_*max*_*/*h	8	0.68±0.18	0.69±0.10	0.80±0.27
*K*_*e*_*/*h^-1^	8	0.87±0.16	0.72±0.14	0.76±0.09

Parameters were calculated from plasma chlorzoxazone concentration profiles (Fig 4) as described in the Materials and methods. Data represent the mean ± SD (n = 6).

*P<0.05 and

**P<0.01 vs the control group.

^#^*P*<0.05 and

^##^*P*<0.01 vs the IM group.

The significance was determined by one-way analysis of variance (ANOVA) followed by Tukey’s test. AUC, area under the curve; T_*1/2*_, half-life; *C*_*max*_, peak concentration; *T*_*max*_, peak time; and *K*_*e*_, elimination rate constant.

**Fig 5:**
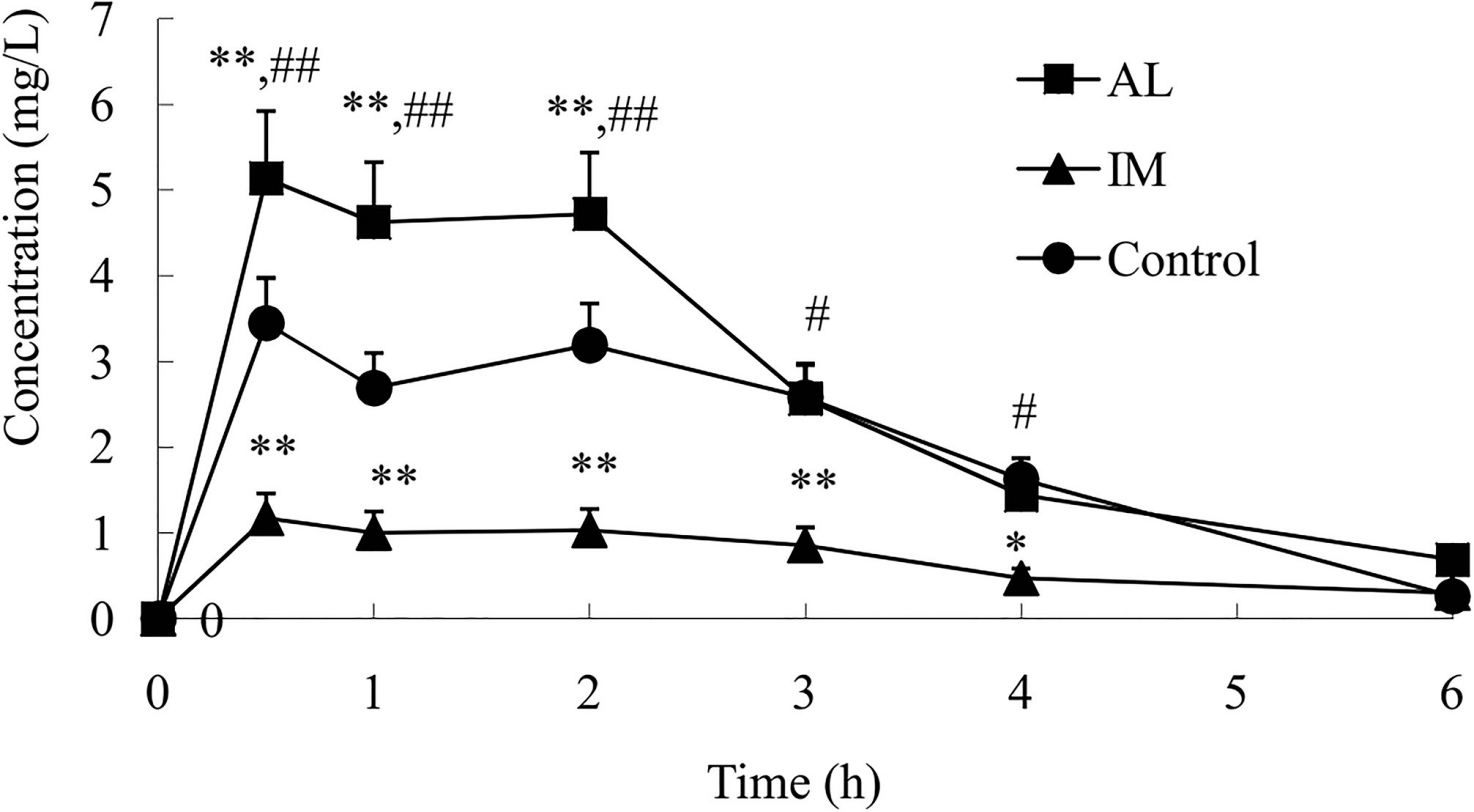
Plasma concentration–time profiles of 6-OH chlorzoxazone after oral administration to control normal saline rats, immune-mediated liver injury rats, and alcohol-induced liver injury rats. Blood samples were collected at 0.5, 1, 2, 3, 4, and 6 h post chlorzoxazone administration (50 mg/kg). Plasma 6-OH chlorzoxazone levels were measured as described in the materials and methods. Data represent the mean ± SD (n = 6). *P<0.05 and **P<0.01 vs the control group. ^#^*P*<0.05 and ^##^*P*<0.01 vs the IM group. The significance was determined by one-way analysis of variance (ANOVA) followed by Tukey’s test.

The mean pharmacokinetic parameters and plasma concentration–time profiles for 6-OHCHZ after pretreatment with BCG and AL are shown in Table 2 and Fig 5, respectively. The 6-OHCHZ/CHZ ratios after pretreatment with BCG and AL are shown in Table 2. BCG pretreatment significantly decreased the 6-OHCHZ/CHZ ratios of *C*_*max*_, AUC, *T*_*1/2*_, and *Ke* to 75%, 80%,10%, and 36%, respectively, compared to those inthe control group, which suggested reduced metabolism of CHZ to 6-OHCHZ. In contrast, AL pretreatment significantly increased the 6-OHCHZ/CHZ ratios of *C*_*max*_, AUC, and *T*_*1/2*_ to 125%, 460%, and 10%, respectively, compared to those in the control group, which suggested induced metabolism of CHZ to 6-OHCHZ. Consequently, the metabolic activity of CYP2E1 was differentially expressed during the development of alcohol-induced and immune-mediated liver injuries in the rat models.

**Table 2:** Thepharmacokinetic parameters of 6-OH chlorzoxazone after a single oral dose of chlorzoxazone (50 mg/kg) in control, IM and AL rats (mean ± SD, *n* = 6).

Pharmacokinetic parameters	control	AL	IM
AUC/h•mg•L^-1^	1.34 ±0.27	4.62 ±0.91**^##^	0.50 ±0.09**
*T*_*1/2*_/h	1.43 ±0.26	1.66 ±0.34^##^	2.56 ±0.53**
*C*_*max*_/mg•L^-1^	3.19 ±0.70	4.72 ±0.92**^##^	1.03 ±0.26**
*K*_*e*_/h^-1^	0.48 ±0.05	0.42 ±0.05^#^	0.27 ±0.04*

Parameters were calculated from plasma 6-OH chlorzoxazone concentration profiles (Fig 5) as described in the Materials and methods. Data represent the mean ± SD (n = 6).

*P<0.05 and

**P<0.01 vs the control group.

^#^*P*<0.05 and

^##^*P*<0.01 vs the IM group.

The significance was determined by one-way analysis of variance (ANOVA) followed by Tukey’s test.

### Changes in NF-κB, iNOS, and CYP2E1 expression

**Fig 6:**
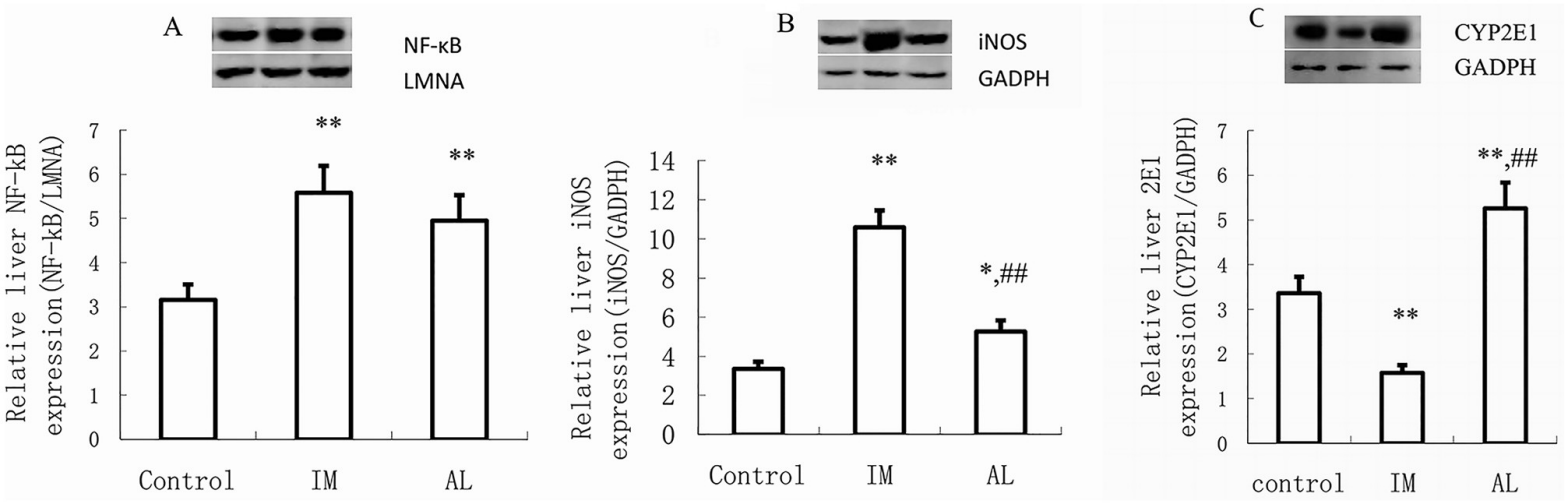
Effect of immune-mediated or alcohol-induced liver injury on NF-κB, iNOS, and CYP2E1 protein levels in rats. The rats were treated with BCG (125 mg/kg, intravenously) and analyzed two weeks later or fed an ethanol-containing diet for 5 weeks as indicated. Liver proteins (nuclear protein was used to detect NF-κB and liver tissue protein was to detect iNOS and CYP2E1) were extracted, and equal amounts (30 μg) of protein were subjected to SDS-PAGE followed by western blot analysis using antibodies specific for NF-κB, iNOS, and CYP2E1. The results were normalized to LMNA or GAPDH. NF-κB (A), iNOS (B), and CYP2E1 (C) protein expression in the rat liver was detected by western blot. NF-κB, iNOS, and CYP2E1 expression was quantified using Image Quant software. Data are presented as the mean ± SD from three independent experiments (n = 9). *P<0.05 and **P<0.01 vs the control group. ^#^*P*<0.05 and ^##^*P*<0.01 vs the IM group. The significance of the data was determined by one-way analysis of variance (ANOVA) followed by Tukey’s test.

Western blot analysis showed that the protein densities in each lanewere uniform relative to those of the internal reference LMNA or GAPDH, which suggested that the protein quantifications were accurate and that the amounts of protein in the samples were consistent. NF-κB protein expression was increased significantly in the IM group compared with that in the control group. Similarly, the amount of protein translocating to the nucleus was significantly increased following alcohol consumption (AL group; Fig 6A). iNOS protein expression was standard in the cytoplasms of normal liver cells and significantly higher in the IM group than in the control group but lower in the AL group than in the IM group (Fig 6B). As shown in Fig 6C, CYP2E1 exhibited basal expression levels in normal liver tissue. BCG injection led to a significant decrease in CYP2E1 expression, whereas alcohol stimulation led to a significant increase in CYP2E1 expression (*P*< 0.05) compared to that in the control group.

### Effects of immune-mediated and alcohol-induced liver injury on TNF-α and IL-1β contents in rats

**Fig 7:**
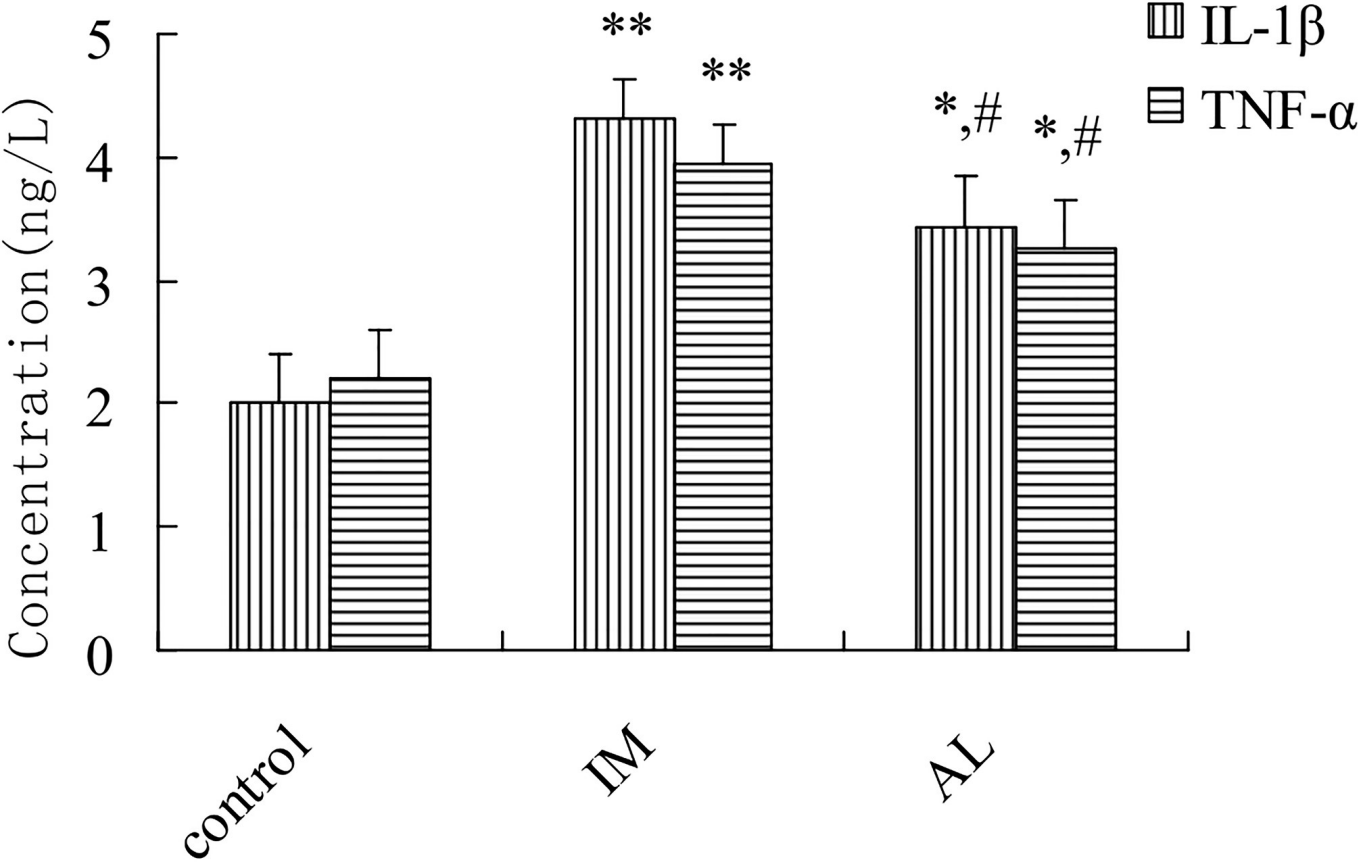
Effect of immune-mediated or alcohol-induced liver injury on the TNF-α and IL-1β contents in rat liver homogenate. The rats were treated with BCG (125 mg/kg, intravenously) and analyzed two weeks later or fed an ethanol-containing diet for 5 weeks as indicated. Each bar represents the mean ± SD from three independent experiments(n = 9/group). *P<0.05 and **P<0.01 vs the NS group. ^#^*P*<0.05 and ^##^*P*<0.01 vs the IM group. The significance of the data was determined by one-way analysis of variance (ANOVA) followed by Tukey’s test.

As shown in Fig 7, when the rats were injected with BCG for 14 days, the levels of the proinflammatory cytokines TNF-α and IL-1β were significantly elevated compared with those in the control group. The TNF-α and IL-1β levels in the AL group were increased slightly compared with those in the control group and decreased significantly compared with those in the IM group (*P*< 0.05).

### Effect of inhibiting NF-κB activation on CYP2E1 mRNA expression in rats with immune-mediated or alcohol-induced liver injury

**Fig 8:**
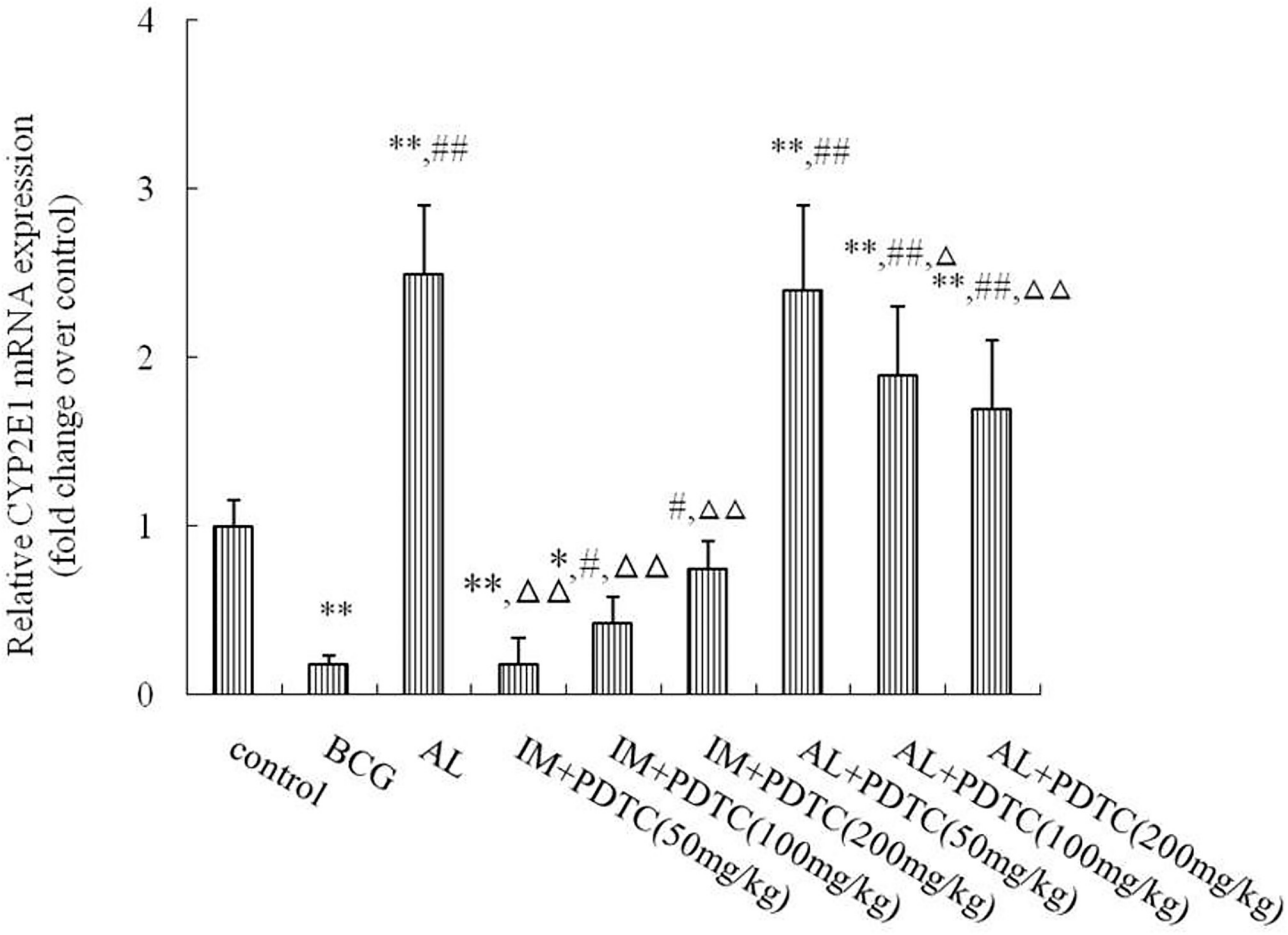
Effect of PDTC on the CYP2E1 mRNA levels in rat liver tissues subjected to immune-mediated or alcohol-induced liver injury. The rats were treated with BCG (125 mg/kg, intravenously) and analyzed two weeks later or fed an ethanol-containing diet for 5 weeks as indicated. Each bar represents the mean ± SD from three independent experiments (n = 9/group). *P<0.05 and **P<0.01 vs the NS group.^#^*P*<0.05 and ^##^*P*<0.01 vs the IM group. ^Δ^*P*<0.05 and ^ΔΔ^*P*<0.01 vs the AL group. The significance of the data was determined by one-way analysis of variance (ANOVA) followed by Tukey’s test.

We found high NF-κB expression in the liver tissues of IM and AL rats. To further explore the effects of NF-κB on CYP2E1 transcription during the two injury processes, we used PDTC to inhibit NF-κB and observe its regulation of CYP2E1 transcription. As shown in Fig 8, the CYP2E1 mRNA levels were significantly decreased in the IM liver samples compared with those in the control group (*P*< 0.01) and significantly increased in the AL liver samples (*P*< 0.01). The CYP2E1 mRNA levels in the AL plus PDTC group were decreased in a dose-dependent manner (ED_50_:76mg/kg, *P*< 0.05), similar to those in the control group. However, their levels in the IM plus PDTC group were increased in a dose-dependent manner (*P*< 0.05), similar to those in the control group.

## Discussion

Morphological experiments confirmed that immune-mediated liver injury was mainly caused by inflammatory reactions, while alcoholic liver injury was mainly caused by oxidative damage. Therefore, the degree of inflammatory reaction was an important mechanism leading to the differences in CYP2E1 in the two liver injuries. Compared with alcohol-induced liver damage, BCG immunostimulation activated more macrophages (Kupffer cells) and other nonspecific immune cells in the liver and could thus induce more endogenous inflammatory cytokines such as TNF-α and IL-1β. These results were consistent with those of other scholars [16–18]. We also observed that necrosis and the collapse of liver lobules in the livers of rats with immune liver injury were also important reasons for the downregulation of CYP2E1 protein expression.

*In vivo* experiments showed that CYP2E1 protein expression regulation was preceded by mRNA regulation, implicating transcription as the primary mechanism. To assess CYP2E1 transcript level regulation, we observed changes in NF-κB and explored the transcriptional regulation of CYP2E1 using the inhibitor PDTC. IL-β and TNF-α could induce iNOS expression in parenchymal hepatic cells or Kupffer cells, which in turn, induced substantial NO production [18]. IL-1β, TNF-α, and iNOS may induce NF-κB over expression to further regulate the liver injury process [19]. Indeed, we found that both oxidative stress and inflammatory reactions could cause liver injury. Interestingly, the two reactions shifted, with one side being stronger, and the other being relatively weak. NF-κB was highly sensitive to both stimuli, leading to almost equal inductions of both liver injury processes. However, when NF-κB was inhibited in both liver injury models, the transcriptional regulation of CYP2E1 was bidirectional. This finding demonstrated that NF-κB serves as a link between oxidative/nitrative stress and immune damage and illustrated the complexity of CYP2E1 transcriptional regulation [20]. In alcohol-induced liver injury, the increased expression and metabolic activity of CYP2E1 as well as the increased oxidative metabolism could generate substantial amounts of reactive oxygen free radicals that induce lipid peroxidation, which could damage the structures and functions of various organelles and enzymes, suppress proteasome activity, decrease acetaldehyde adduct proteolysis, and damage liver cells [21,22]. Inimmune-mediated liver injury, CYP2E1 was downregulated, and CYP2E1-catalyzed oxidative metabolism was weakened. However, excessive iNOS, which catalyzes the guanidino nitrogen on L-arginine into NO [23], was generated, thereby inducing strong nitrative stress and damaging the cell membrane structure, resulting in liver injury [24]. Our study also found that the downregulation of CYP2E1 in immunological liver injury was NO-dependent, and inhibition of iNOS relieved inflammatory liver cell infiltration and dose-dependently inhibited the transcriptional downregulation of CYP2E1; this result was consistent with previous reports in the literature [25]. However, iNOS induction was not significant in alcoholic liver injury, which was slightly different from the literature [26, 27] and may have been caused by the different models. Tindberg et al.[28] also described an NF-κB binding site in intron 2 of the CYP2E1 gene that functions as a cytokine-sensitive repressor in rat primary cortical glial cell cultures. According to our experiments and the above literature, we speculated that during immune liver injury, the transcriptional downregulation of CYP2E1 via the NF-κB/IL-β, TNF-α pathway and high expression of iNOS resulted from the high expression of downstream inflammatory cytokines such as IL-β and TNF-α after NF-κB activation. Moreover, iNOS further stimulated NF-κB expression, forming a vicious circle and aggravating the process of liver injury. In addition to NF-κB, iNOS could also directly act on the iron-sulfur reaction center of CYP2E1 and destroy the protein, which may be a reason for its downregulated expression [29]. Furthermore, iNOS is an important molecular source of nitration in CYP2E1 posttranslational protein modification [20, 24], explaining why the decreased metabolic activity in immune liver injury was greater than the protein expression.

In addition, CYP2E1 is also regulated at the posttranscriptional and posttranslational levels [30]. In the process of alcoholic liver injury, a large amount of insulin is induced, and insulin directly downregulates the gene expression of CYP2E1 by reducing the mRNA stability [30, 31]. During the process of immunological liver injury, due to the large amount of inflammatory cytokines and iNOS, the CYP2E1 protein is phosphorylated, nitrated and ubiquitinated. These effects ultimately alter the metabolic activity of CYP2E1 [32]. In addition, other scholars have studied JNK was also regulating CYP450 in extra-hepatic tissues, which may serve as a secondary mechanism for the regulation of CYP2E1 [33]. These experiments will be carried out at the subsequent cell level, and a substantial amount of work must be performed to elucidate the regulatory mechanism of CYP2E1.

In conclusion, the differential changes in CYP2E1 during the two different liver injury processes resulted from different transcriptional regulatory pathways. NF-κB activation during the process of alcohol-mediated liver injury might be caused by drug-metabolizing enzyme-induced oxidative stress, whereas IL-1β, TNF-α, and iNOS might be the primary NF-κB-inducing factors during immune-mediated liver injury. Therefore, we speculate that NF-κB may be an important link among oxidative stress, nitrative stress, and inflammatory responses. The differential changes in CYP2E1 associated with the aforementioned liver injury types might be attributed to self-regulatory processes in liver cells as they adapt to environmental stimuli. Therefore, abnormalities in CYP2E1 might also be considered a diagnostic indicator of abnormal liver function. Further studies are warranted to determine whether liver injury processes can be inhibited by regulating CYP2E1 via NF-κB interference, whether CYP2E1 can be selectively regulated to interfere with the injury process and the underlying mechanisms. Additionally, more work is needed to determine whether this mechanism also applies to other P450 enzymes.

## Supporting information

S1 Fig Changes in NF-κB expression (Western blot analysis, Fig 6A). (PDF)

S2 Fig Changes in iNOS expression (Western blot analysis, Fig 6B). (PDF)

S3 Fig Changes in CYP2E1 expression (Western blot analysis, Fig 6C). (PDF)

S4 Fig Changes in GADPH expression (Western blot analysis. Fig 6). (PDF)

S1 Minimal data set Contains minimal data set for the following files: Fig3F. Histopathological effects of inhibiting iNOS activation in rats with immune liver injury. Fig3G. Histopathological effects of inhibiting iNOS activation in rats with immune liver injury. Fig4. Plasma concentration–time profiles of chlorzoxazone (50 mg/kg) after oral administration to normal saline control rats, immune-mediated liver injury rats, and alcohol-induced liver injury rats. Fig5. Plasma concentration–time profiles of 6-OH chlorzoxazone after oral administration to control normal saline rats, immune-mediated liver injury rats, and alcohol-induced liver injury rats. Fig6. Effect of immune-mediated or alcohol-induced liver injury on NF-κB, iNOS, and CYP2E1 protein levels in rats. Fig7. Effect of immune-mediated or alcohol-induced liver injury on the TNF-α and IL-1β contents in rat liver homogenate. Table 1. The pharmacokinetic parameters of chlorzoxazone after a single oral dose (50 mg/kg) in control, IM, and AL rats (50 mg•L-1, mean ± SD, n = 6). Table 2. Thepharmacokinetic parameters of 6-OH chlorzoxazone after a single oral dose of chlorzoxazone (50 mg/kg) in control, IM and AL rats (mean ± SD, n = 6).
(XLSX)
