# Supplementary figures and images for: NF-κB-mediated regulation of rat CYP2E1 by two independent signaling pathways
Source: PLoS One. 2019 Dec 27;14(12):e0225531. doi: 10.1371/journal.pone.0225531 (PMC6934272; doi:10.1371/journal.pone.0225531)

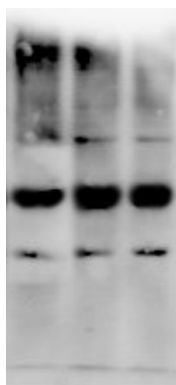

S1 Fig. Changes in NF- $\kappa$ B expression (Western blot analysis, Fig 6-A)

Supplement: S1 Fig — (PDF) [file pone.0225531.s001.pdf]

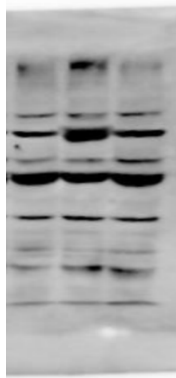

S2 Fig. Changes in iNOS expression (Western blot analysis, Fig 6-B)

Supplement: S2 Fig — (PDF) [file pone.0225531.s002.pdf]

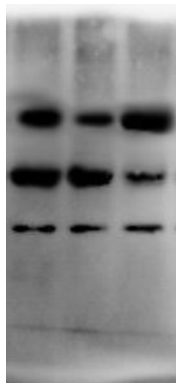

S3 Fig. Changes in CYP2E1 expression (Western blot analysis, Fig 6-C)

Supplement: S3 Fig — (PDF) [file pone.0225531.s003.pdf]

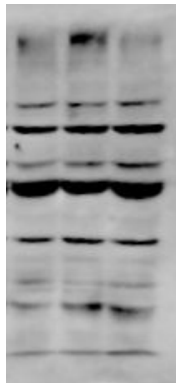

S4 Fig. Changes in GADPH expression (Western blot analysis. Fig 6)

Supplement: S4 Fig — (PDF) [file pone.0225531.s004.pdf]
